# Leptospirosis Outbreak following Severe Flooding: A Rapid Assessment and Mass Prophylaxis Campaign; Guyana, January–February 2005

**DOI:** 10.1371/journal.pone.0039672

**Published:** 2012-07-09

**Authors:** Amy M. Dechet, Michele Parsons, Madan Rambaran, Pheona Mohamed-Rambaran, Anita Florendo-Cumbermack, Shamdeo Persaud, Shirematee Baboolal, Mary D. Ari, Sean V. Shadomy, Sherif R. Zaki, Christopher D. Paddock, Thomas A. Clark, Lazenia Harris, Douglas Lyon, Eric D. Mintz

**Affiliations:** 1 Centers for Disease Control and Prevention, Atlanta, Georgia, United States of America; 2 Portland Providence Medical Center, Portland, Oregon, United States of America; 3 Georgetown Public Hospital Corporation, Georgetown, Guyana; 4 Ministry of Health, Georgetown, Guyana; 5 Caribbean Epidemiology Centre, Port of Spain, Trinidad and Tobago; 6 Centers for Disease Control and Prevention, Georgetown, Guyana; 7 La Clinica del Cariño, Hood River, Oregon, United States of America; University of Minnesota, United States of America

## Abstract

**Background:**

Leptospirosis is a zoonosis usually transmitted through contact with water or soil contaminated with urine from infected animals. Severe flooding can put individuals at greater risk for contracting leptospirosis in endemic areas. Rapid testing for the disease and large-scale interventions are necessary to identify and control infection. We describe a leptospirosis outbreak following severe flooding and a mass chemoprophylaxis campaign in Guyana.

**Methodology/Principal Findings:**

From January–March 2005, we collected data on suspected leptospirosis hospitalizations and deaths. Laboratory testing included anti-leptospiral dot enzyme immunoassay (DST), immunohistochemistry (IHC) staining, and microscopic agglutination testing (MAT). DST testing was conducted for 105 (44%) of 236 patients; 52 (50%) tested positive. Four (57%) paired serum samples tested by MAT were confirmed leptospirosis. Of 34 total deaths attributed to leptospirosis, postmortem samples from 10 (83%) of 12 patients were positive by IHC. Of 201 patients interviewed, 89% reported direct contact with flood waters. A 3-week doxycycline chemoprophylaxis campaign reached over 280,000 people.

**Conclusions:**

A confirmed leptospirosis outbreak in Guyana occurred after severe flooding, resulting in a massive chemoprophylaxis campaign to try to limit morbidity and mortality.

## Introduction

Leptospirosis is a zoonosis caused by pathogenic species of *Leptospira*. It is usually transmitted through skin or mucus membrane contact with water or soil contaminated with urine from infected animals, or through contact with tissues from infected animals. While the majority of infections are subclinical or mildly symptomatic, leptospirosis can result in severe symptoms or even death, with mortality rates ranging from 5%–50% [1], [2], [3].

Leptospirosis is considered to be widespread in many tropical countries, including the Caribbean region and Central and South America [4], [5], [6], and outbreaks have occurred after severe flooding due to increased contact with contaminated water [7], [8], [9]. In Guyana, leptospirosis has been detected in humans and livestock, but prior to 2005, no outbreaks had been reported [4], [10], [11], [12]. Antimicrobial chemoprophylaxis with weekly doxycycline has been found to be protective against clinical leptospirosis during outbreaks or high levels of water exposure and may even reduce mortality [13], [14], [15], although conclusive evidence for this is still lacking [16].

On January 15, 2005, after a month of unusually high rainfall in Guyana, 10 inches of rain fell in 15 hours, leading to extensive flooding along the densely populated Atlantic coast where most of the population resides. Over 300,000 of Guyana’s 750,000 inhabitants were affected, and an estimated 70,000 were displaced. Water began to recede in Georgetown, the capital city, by January 20, but in many coastal areas flooding lasted for several weeks.

On January 24, the Guyana Ministry of Health (MOH) requested assistance from the United States Centers for Disease Control and Prevention (CDC) to enhance surveillance for waterborne disease [5]. On January 29, a previously healthy 28-year-old man died of liver failure and refractory hypotension. In the following two days, seven more individuals succumbed with similar symptoms. Experienced clinicians and pathologists were concerned that these deaths and other hospitalizations were due to leptospirosis, but no diagnostic tests were available for laboratory confirmation. Surveillance activities for waterborne disease were subsequently modified to include detection and evaluation for leptospirosis. A case series related to this outbreak has been published [17].

Given the potential for widespread transmission of leptospirosis and significant morbidity and mortality, immediate action was necessary to confirm the etiology and extent of the outbreak and to initiate preventive measures. Here we briefly describe the epidemiologic investigation and the rapid public health response to the outbreak, including a massive chemoprophylaxis campaign.

## Methods

### Ethics Statement

This investigation was conducted as part of an emergency public health response and was not considered to be research. As such, it was not subject to Investigational Review Board (IRB) review requirements by the CDC. All organizations actively involved with the investigation, including the local hospitals, the MOH, the Pan American Health Organization (PAHO), and the Caribbean Epidemiology Centre (CAREC), agreed that IRB review was not necessary. Verbal informed consent was obtained from all patients or family members before conducting interviews or obtaining specimens for clinical testing. Patients were given the opportunity to refuse and, if they did so, did not see a change in their medical care. Written consent was not possible due to logistical limitations of multiple testing and survey site locations and the need for immediate data collection in the setting of the outbreak.

### Case Definitions

As the standard CDC case definition for leptospirosis is based on laboratory tests that were not widely available during this outbreak (e.g. microscopic agglutination test (MAT) or tissue staining) [18], we used the following alternative case-definitions. We defined a suspected case of leptospirosis as a patient with signs and/or symptoms consistent with leptospirosis as determined by the healthcare provider in the absence of laboratory testing, or in the presence of a negative or indeterminate IgM dot-ELISA test (Dip-S-Ticks (DST), PanBio® Inc. Columbia, MD [19]. We defined a probable case of leptospirosis as a patient with signs and/or symptoms consistent with leptospirosis and a positive result on a single serum sample by either the IgM dot-ELISA, or the MAT (a single Leptospira agglutination titer ≥800), or both [20]. We used the CDC case definition for a confirmed case: a patient with a fourfold or greater increase in *Leptospira* agglutination titer between acute and convalescent phase serum specimens obtained up to 14 days apart and studied at the same laboratory and/or *Leptospira* demonstrated by immunohistochemical tissue staining (IHC) [21], [22]. Suspected, probable, and confirmed leptospirosis deaths were defined as deaths among patients who met the respective case definitions.

### Patient Information

We collected basic demographic information on all patients admitted to Georgetown Public Hospital Corporation (GPHC) with suspected leptospirosis from January 25–March 3, 2005 after obtaining consent. We acquired reports of all deaths through active mortality surveillance of hospitals, morgue registries, mobile clinics, death certificates, media news reports, and social services assisting with burial activities. All deaths reported through these channels were investigated and attempts were made to determine whether or not the death was due to leptospirosis based on clinical presentation and available laboratory testing. Beginning February 7, staff from the MOH implemented a standardized questionnaire focusing on symptoms and exposures among inpatients and outpatients with suspected leptospirosis evaluated from January 26–February 21, 2005 at GPHC and four other medical facilities that served areas affected by flooding.

### Laboratory Testing

On February 2, staff at the Central Medical Laboratory (CML) in Georgetown was trained by CDC investigators in DST test kit use. The MAT was performed on a subset of samples at the CDC using a panel of 20 individual serovars (Australis, Bratislava, Autumnalis, Ballum, Bataviae, Canicola, Celledoni, Cynopteri, Djasman, Grippotyphosa, Borincana, Iceterohaemorrhagiae, Mankarso, Javanica, Georgia, Alexi, Pomona, Pyrogenes, Wolffi, Tarassovi) [23]. Kidney, liver, lung, central nervous system, heart, and spleen tissue samples from 12 patients whose deaths were attributed to leptospirosis were sent to CDC for IHC staining for detection of leptospira; however, not all of these tissues were submitted for each patient [21], [22]. Due to the requirement for specialized media for culture of leptospirosis, which was not available to MOH and CDC investigators at the time of the outbreak investigation, cultures for leptospirosis were not be obtained. At the time of the outbreak, no assay for the diagnosis of leptospirosis by polymerase chain reaction (PCR) was approved, so this test was not employed.

## Results

### Case Surveillance at GHPC

From January 25 to March 3, 2005, 236 patients were admitted to GPHC with suspected leptospirosis (median age 32, 57% female) (Figure S1). The peak number of admissions (26) occurred on February 3. Of the 236 patients admitted, 105 (44%) were tested with Dip-S-Tick IgM ELISA; 52 (50%) were positive, 41 (39%) negative, and 12 (11%) indeterminate. Ultimately, based on additional test results, 2 of these 105 patients were confirmed, 53 probable, and 50 suspected cases of leptospirosis.

Single specimens from 19 cases and paired specimens from 7 cases were tested by MAT at CDC. All five samples that tested negative by DST at CML were negative by MAT at CDC. Six of the 14 single specimens that tested positive by DST at CML were compatible with probable leptospirosis with titers ≥800, and four had titers <800. The remaining four specimens were negative by MAT, but one of these was IHC positive postmortem. Convalescent serum was not available for these cases. Of the seven patients with paired sera, four were confirmed leptospirosis, demonstrating a four-fold rise in titer by MAT. Six of these seven serum pairs had at least one sample with a titer ≥800, including two that had samples with titers ≥1600 but that did not show a four-fold rise. The serovars most frequently represented by the 12 samples with titers ≥800 from distinct patients included Icterohaemorrhagiae (11, 92%), Mankarso (11, 92%), Georgia (10, 83%), and Bratislava (8, 67%). The most common serovars representing a four-fold rise in titer by MAT were Icterohaemorrhagiae (4, 100%), Mankarso (4,100%), Georgia (4, 100%), Bratislava (4, 100%), Autumnalis (3, 75%), and Cynopteri (3, 75%).

### Mortality

A total of 34 deaths were recorded during the investigation. Eleven deaths were laboratory confirmed as leptospirosis (ten by IHC and one by four–fold rise in MAT titer), ten were probable leptospirosis deaths, and 13 were suspected leptospirosis deaths (ten patients without serum or tissue available for testing, one negative by IHC, and two DST negative, one of whom additionally tested negative by IHC). In total, liver and/or kidney tissue specimens from 10 of 12 patients evaluated by IHC staining were positive for *Leptospira.* One of the IHC negative patients was a 32-year-old man with significant flood water exposure who was admitted to the hospital with fever, jaundice, and myalgias and developed renal failure and hemorrhage before death. Rapid testing for leptospirosis was negative, but subsequent IHC evaluation for *Plasmodium falciparum* showed extensive intraerythrocytic infection with this parasite in multiple tissues [24].

### Inpatient and Outpatient Questionnaire

The standardized questionnaire was successfully administered to 201 patients. The most common symptoms were fever, headache, vomiting, and myalgia (Table S1). Nearly all (89%) of the 201 interviewed patients reported daily contact with flood water. Contact with animals since the onset of the flood was reported by 64%. Dogs and rats were most frequently mentioned. Fifty-four percent of individuals noted animals in their home not present before the flood, including rats (79%), dogs (24%), and cats (14%). There was no significant difference in reported flood water-contact time (median 1–3 hours) or in the types of animals with which patients had contact among those with confirmed, probable, or suspected leptospirosis (data not shown). Overall, 37 (39%) of the 95 interviewed patients with information on chemoprophylaxis administration received chemoprophylaxis before hospital presentation; the proportion was slightly lower in patients who tested DST-positive (24%) than those who tested DST-negative (31%), but this difference did not reach statistical significance.

The 17 patients interviewed before their deaths were not more likely than those who survived to report a delay in hospitalization from onset of symptoms, longer contact times with flood waters, or contact with any particular type of animal.

### Chemoprophylaxis Campaign

Shortly after flooding occurred, nightly meetings were held to discuss emergency response efforts with representation from the Ministry of Health of Guyana, the CDC, PAHO, CAREC, and other organizations. Preparations were made for a chemoprophylaxis campaign based on the occurrence of multiple deaths suspected to be due to leptospirosis starting on January 29 and on the consensus of the group in determining that such an intervention might help limit morbidity and mortality. Possible negative effects of chemoprophylaxis were discussed and benefits were believed to outweigh harm. A government-owned local pharmaceutical company, already supplying doxcycline to medical facilities in the country, was asked to convert all efforts to doxcycline production. This allowed for initiation of a massive chemoprophylaxis campaign, funded by the Ministry of Health of Guyana, to begin on February 2, 2005, the day after the first case of leptospirosis was confirmed. Efforts were made to administer a weekly 200 mg dose of doxycycline to an estimated 300,000 persons exposed to flood waters. Children younger than 8 years old, pregnant women, and breast-feeding mothers received a weekly 5-day course of amoxicillin instead of doxycycline during the campaign.

Each morning health department workers and volunteers gathered at the Ministry of Health building where the ground floor was converted to an emergency operations supply center. People were assigned to travel via pick-up truck with supplies of doxycycline to specific locations of the flood-affected areas, including thirteen semi-permanent stationary sites, established in community buildings, churches, health centers, schools, and employment facilities, and up to 23 roaming sites via labeled vans parked in prominent areas of the community. Hospitals served as additional permanent distribution centers. Amoxicillin was available only in hospitals and health centers. Citizens were alerted to the need for prophylaxis through public health messages via radio, television, newspapers, loudspeakers, and postings and were encouraged to report to one of the distribution areas. When possible, directly observed therapy was utilized. If medical personnel were informed of individuals who were unable to present in person, a delivery of medications was made to the home. If, upon presentation to a distribution site or during a home visit, volunteers were concerned about possible illness, patients were referred or transported to a health center or a hospital for further evaluation.

On the first day of the chemoprophylaxis campaign, 22 field teams consisting of 112 volunteers were able to administer all 500 available doses of doxycycline, As supplies increased, each day more doses were delivered. An estimated 280,000 people received doxycycline prophylaxis by the end of the first week, 250,000 by the end of the second week, and 85,000 by the end of the third week. The target population during the third week was limited to persons inhabiting areas where flood waters were still present. Chemoprophylaxis was delivered weekly rather than in a single distribution with 3 weeks’ supply because of initial medication shortage and uncertainty as to the anticipated duration of the flooding.

On the day the chemoprophylaxis campaign began, 18 patients were admitted to GPHC with suspected leptospirosis with peak admissions (26) occurring the following day. Over the next 3 weeks the number of admissions declined, and by February 22, the last day of the campaign, only one patient was admitted with suspected leptospirosis.

## Discussion

Widespread flooding in Guyana led to conditions favorable for epidemic leptospirosis. Once clinical and pathological recognition raised concerns that early febrile illness cases and deaths were the result of leptospirosis, the Guyana MOH rapidly recognized the need to adapt emergency waterborne disease surveillance to detect and monitor an emerging leptospirosis outbreak. Because of this transition, standard case definitions were modified to accommodate the situation in this post-disaster environment. In collaboration with national, regional, and international organizations, the MOH quickly obtained diagnostic capacity in-country and launched a massive chemoprophylaxis campaign. These actions likely assured appropriate treatment for clinical cases and may have prevented additional cases from occurring.

The effectiveness of weekly chemoprophylaxis in the prevention of leptospirosis infection during outbreaks in endemic areas is questionable; however, weekly chemoprophylaxis has been shown to reduce severity of clinical illness and potentially to reduce mortality during seasonal outbreaks or following high levels of water exposure, even though serologic evidence of infection did not differ in those who received doxycycline versus those who received placebo [13], [14]. When used in United States soldiers in Panama, weekly doxycycline demonstrated a protective efficacy of 95% (p<0.001) for infection [15]. Decision tree analysis of the cost effectiveness of empirical antimicrobial prophylaxis and treatment of leptospirosis showed that in regions with both high and low background incidence rates for leptospirosis, doxycycline prophylaxis, as compared to a no-prophylaxis strategy, provided cost savings, decreased severity of illness and mortality, and improved health outcomes [25].

In this outbreak, it is difficult to assess the efficacy of the prophylaxis campaign. As Figure S1 demonstrates, the number of cases decreased after the administration of prophylaxis to the community. However, during this same time period public awareness increased, flood waters began to recede, and the epidemic may have run its course. Additionally, this outbreak follows a similar time course and distribution pattern to other leptospirosis outbreaks where no chemoprophylaxis was delivered [7], [26], [27], [28]. Of the surveyed patients, a significant number who had received doxycycline chemoprophylaxis had positive DST testing, although the number was lower than those who had not. This could result from DST testing detecting serologic evidence of prior infection and not active infection and illness, administration of doxycycline after infection and illness were already present, or lack of effectiveness of the medication to prevent illness. A placebo-controlled trial would have been scientifically more rigorous, but was precluded by concerns based on studies published prior to this outbreak that had demonstrated benefit in providing prophylaxis [13], [14], [15] and by the logistical and political complexities of implementing a research component into an emergency response when public distress was at its highest. The effectiveness of this chemoprophylaxis campaign could have been assessed via a serologic survey to determine the proportion of people who received doxycycline and had evidence of symptomatic or asymptomatic seroconversion; however, resource limitations did not permit this to occur.

With the lack of definitive data to guide decisions about initiating a chemoprophylaxis campaign, the Guyana experience does not suggest that chemoprophylaxis should be initiated without thorough consideration. Certainly the potential benefit of massive chemoprophylaxis has to be weighed against the potential for drug resistance. While there has been no documentation of *Leptospira* acquiring resistance of which the authors are aware, other bacteria may do so when exposed to antibiotics. Ideally prophylaxis is undertaken when point-source exposure is known and at-risk individuals can be targeted, such as in smaller outbreaks. In times of severe flooding and in developing countries, identifying and providing chemoprophylaxis only to those individuals who will develop leptospirosis is not feasible; therefore far larger numbers of patients will need to be treated in order to derive maximal individual and public health benefit. Should situations in the future warrant a chemoprophylaxis campaign such as was undertaken in Guyana because of the large number of at-risk individuals and the potential for widespread disease and fatalities, a case control study or other scientifically rigorous evaluation of its efficacy should be strongly considered. The logistics of a large-scale chemoprophylaxis campaign should not necessarily be considered insurmountable, as is highlighted by the successful delivery of medication to over 280,000 individuals in the immediate aftermath of severe flooding in Guyana.

Given the mode of transmissibility of leptospirosis through animal urine, it is not surprising that many patients reported exposure to animals. Those depending on their livestock for financial reasons disclosed bringing their animals into the home in order to prevent the animals’ death or loss. Rats in the home were a common complaint. Through public messaging, people were encouraged to try to eliminate rat entry by removing garbage, but due to the limited time and financial resources during the outbreak, no specific rodent control programs could be initiated. With the foresight afforded by this outbreak and response, future public health interventions, such as chemoprophylaxis campaign strategies, or implementation of disease prevention measures such as rodent control programs, can be evaluated for their efficacy at preventing exposure and illness.

Steps taken in response to the leptospirosis outbreak after the flood have yielded longer-term benefits. Clinicians and the general public in Guyana are now more aware of the disease and the means by which it is spread and can be prevented. This heightened awareness, and the availability of diagnostic testing in-country, will improve surveillance for sporadic cases of leptospirosis and help prevent, detect, and control future outbreaks. In the aftermath of this outbreak in 2005, these conditions were set in place in Guyana and allowed for prompt recognition and response to a similar leptospirosis outbreak occurring the following year [29].

The danger of the heightened sensitivity to a particular disease in an outbreak setting is the tendency to overlook other diseases that may have similar clinical presentations. In this outbreak, previous experience led to initial concerns about waterborne enteric disease, although the ecological conditions were favorable for a leptospirosis outbreak. Furthermore, the individual who was treated for presumed leptospirosis and ultimately succumbed to what was determined to be fulminant malaria based on post-mortem tissue examination, illustrates the need to keep the differential diagnosis open. In the aftermath of severe flooding, epidemics of febrile illnesses spread by mosquitoes, such as malaria and dengue, and by contaminated food and water, such as typhoid fever, may also occur [30], [31], [32]. Distinguishing these diseases on clinical grounds alone can be challenging, and the appropriate treatment and public health interventions vary greatly [26]. Providers were reminded through hospital alerts to evaluate for these diseases concurrently, but focus on the current outbreak did not always result in complete testing, and reliable and rapid results were not always available. Therefore, in order to recognize quickly, assess accurately, and respond appropriately to epidemics of infectious diseases in the post-disaster period, it is essential that rapid and accurate laboratory diagnostic services are available, that public health authorities and health care providers maintain a heightened index of suspicion, and that a timely, representative, and accurate disease surveillance system exists.

Commercially available diagnostic tests for leptospirosis that can be used in the field setting have been evaluated by the CDC [19]. Although an antibody response may not be detected until 10 days or more after initial symptom onset, the use of such assays may help guide appropriate delivery of antimicrobial therapy, which has been shown to reduce the severity and duration of clinical illness, and reduce mortality [33]. The performance of other clinical diagnostic tests which are commercially available or are research-only at this time, including the IgM-ELISA based on the rLipL32/1-LipL21-OmpL1/2 fusion protein [34], has not yet been evaluated in the outbreak setting.

Our investigation of this outbreak and the public health response had several limitations. In the emergency setting, information had to be gathered quickly and was therefore sometimes incomplete. Patient interviews and laboratory testing were not well coordinated, and linking the data later on proved difficult because a centralized identification scheme was not established early. No specific clinical, laboratory, or epidemiologic criteria were required for the case definition of suspected leptospirosis; instead, that determination was made entirely by individual healthcare providers. As awareness of the outbreak increased, the threshold for considering leptospirosis as a diagnosis was lowered and more individuals with suspected leptospirosis were reported than might have occurred had a standard case definition been applied. On the other hand, since leptospirosis often causes only mild or no symptoms, many more patients with leptospirosis were probably treated as outpatients or did not seek medical care. Limited resources precluded laboratory testing of all patients with suspected leptospirosis.

The laboratory tests also have inherent limitations. The DST test has high sensitivity (94.5%) and can detect antibodies as early as 3 days after onset of symptoms; however, the detection rate is generally low early in the disease and antibodies may remain detectable for up to one year [19]. Those tested too early may have had false negative results. Similarly, low titers on MAT may be seen soon after onset, before titers have a chance to rise, or could represent previous exposure rather than new disease. In a well-publicized epidemic such as this, people may have sought medical care early in the course of disease before laboratory confirmation was possible. Culturing blood for leptospirosis would have assisted with confirmation of the outbreak but was not performed. This is a significant limitation to this outbreak investigation, as identification of the infecting serovar or serovars would have aided in identification of the animal reservoirs which contributed to the outbreak. However, with the recognition that leptospirosis cases and outbreaks can occur in Guyana with a greater frequency than previously recognized, preparatory steps can be taken to ensure in future investigations that cultures are obtained and infecting serovars identified in order to guide leptospirosis intervention and control programs.

Despite these limitations, valuable information has been gleaned from the response to this disaster. Natural disasters related to sudden geological and meteorological events are predicted to occur more often as a result of global warming [21]. Human populations are increasingly concentrated in coastal areas that are at high risk for flooding and severe damage resulting from natural disasters. Consequently, the public health community must remain prepared for the sudden emergence of epidemics of infectious diseases like leptospirosis in the post-disaster period. The ability to rapidly detect, confirm, and respond to such infectious disease epidemics in this setting requires an alert, well-coordinated, and well-funded public health system at the local, national, regional and global levels, along with appropriate clinical diagnostic and investigation tools to identify specific etiologic causes. We should take encouragement from the response in Guyana and from other recent successes, but must not relax our efforts to build a strong and well-coordinated public health system worldwide and to improve the accuracy and availability of rapid diagnostic tests that function well under harsh field conditions. Prevention of illness and death in the post-disaster period anywhere in the world will depend on it [35].

## Supporting Information

Figure S1 Cases: Gray: Suspected; Grid Pattern: Probable; Black: Confirmed
(TIF)

Table S1 Symptoms, signs, and laboratory results of hospitalized leptospirosis patients, Guyana, January 26–February 21, 2005.
(DOC)
