# Supplementary figures and images for: Leptospirosis Outbreak following Severe Flooding: A Rapid Assessment and Mass Prophylaxis Campaign; Guyana, January–February 2005
Source: PLoS One. 2012 Jul 9;7(7):e39672. doi: 10.1371/journal.pone.0039672 (PMC3392270; doi:10.1371/journal.pone.0039672)

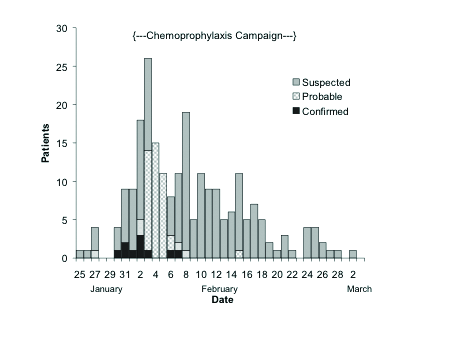

Supplement: Figure S1 — Cases: Gray: Suspected; Grid Pattern: Probable; Black: Confirmed (TIF) [file pone.0039672.s001.tif]
