# Supplementary material for: Leptospirosis Outbreak following Severe Flooding: A Rapid Assessment and Mass Prophylaxis Campaign; Guyana, January–February 2005
Source: PLoS One. 2012 Jul 9;7(7):e39672. doi: 10.1371/journal.pone.0039672 (PMC3392270; doi:10.1371/journal.pone.0039672)
Supplement: Table S1 — Symptoms, signs, and laboratory results of hospitalized leptospirosis patients, Guyana, January 26–February 21, 2005. (DOC) [file pone.0039672.s002.doc]

**Table S1. Symptoms, signs, and laboratory results of hospitalized leptospirosis patients, Guyana, January 26--February 21, 2005.**

|  | Total  (N=201) (n, %) | Confirmed*  (n=7) (n, %) | Probable**  (n=48) (n, %) | Suspected***  (n=146) (n, %) |
| --- | --- | --- | --- | --- |
| Fever | 156 (78) | 6 (86) | 32 (67) | 118 (81) |
| Headache | 116 (58) | 2 (29) | 25 (52) | 89 (61) |
| Vomiting | 109 (54) | 4 (57) | 30 (63) | 75 (51) |
| Myalgia | 106 (53) | 5 (71) | 27 (56) | 74 (51) |
| Abdominal pain | 74 (37) | 3 (43) | 21 (44) | 50 (34) |
| Diarrhea | 61 (30) | 2 (29) | 18 (38) | 41 (28) |
| Conjunctival suffusion | 36 (18) | 2 (29) | 11 (23) | 23 (16) |
| Jaundice | 32 (16) | 3 (43) | 15 (31) | 14 (10) |
| Lethargy | 18 (9) | 1 (14) | 3 (6) | 14 (10) |
| Hemorrhage | 10 (5) | 0 (0) | 7 (15) | 3 (2) |
| Hypotension | 6 (3) | 2 (29) | 2 (4) | 2 (1) |
| Died | 17 (8) | 5 (71) | 9 (19) | 3 (2) |
| DST Tested (N, % positive) | 90 (61) | 7 (100) | 48 (100) | 35 (0) |

- *Confirmed leptospirosis = a patient with a fourfold or greater increase in Leptospira agglutination titer between acute and convalescent phase serum specimens obtained 2 weeks apart and studied at the same laboratory and / or Leptospira demonstrated by immunohistochemical tissue staining (IHC)
- **Probable leptospirosis = patient with suspected leptospirosis and a positive result from either the IgM dot-ELISA (Dip-S-Tick (DST), PanBio® Inc.) or the microscopic agglutination test (a single Leptospira agglutination titer ≥ 800) or both
- *** Suspected leptospirosis based on clinical judgment by the treating healthcare provider in the absence of laboratory testing, or in the presence of a negative IgM dot-ELISA (DST) test.
